# Birth preparedness and its association with place of delivery among women in rural and urban communities of Ogun east senatorial district Nigeria

**DOI:** 10.4314/ahs.v24i2.23

**Published:** 2024-06

**Authors:** Ngozi O Adefala, Temitope Ashipa, Kolawole J Sodeinde, Fikayo E Bamidele, Adebola Y Omotosho, Abiodun O Osinaike, Chimaobi C Nwankpa

**Affiliations:** 1 Department of Community Medicine, Babcock University Teaching Hospital, Ilishan-remo, Ogun State, Nigeria; 2 Department of Obstetrics and Gynaecology, Babcock University Teaching Hospital, Ilishan-remo, Ogun State, Nigeria

**Keywords:** Birth preparedness, practice, place of delivery, utilization of skilled birth attendance

## Abstract

**Background:**

Birth preparedness promotes the timely use of skilled maternal and neonatal care, reduces delays in receiving care; reduces maternal death, and ensures women have professional delivery thus reducing obstetric complications. Making the right decisions regarding the place of delivery influences the outcome of labour and childbirth.

**Objectives:**

To assess the practice of birth preparedness and its association with the place of delivery among women in rural and urban communities of Ogun East Senatorial District.

**Methods:**

A comparative cross-sectional study was carried out among 750 women in the rural and urban communities selected using a multistage sampling technique. An interviewer-administered, structured questionnaire adapted from the safe motherhood questionnaire of the Johns Hopkins Program for International Education in Gynecology and Obstetrics (JHPIEGO) and the Nigeria Demographic Health Survey (NDHS) 2018 was used. Data was analyzed using IBM SPSS version 22.0 and the statistical significance was set at p<0.05. Relevant descriptive and inferential statistics were calculated and results were presented in frequency tables.

**Results:**

Urban respondents were older (mean age 31.07±6.115 years) than their rural counterparts (mean age 30.69±6.312 years). The difference in the mean ages was not statistically significant (p=0.401). Urban respondents were significantly better prepared during their last pregnancy than rural respondents (p=0.022). The majority of respondents in both rural (n=288, 76.8%) and urban areas (n=296, 78.9%) utilized health facilities as a place of delivery during their last pregnancy; the difference was not statistically significant.

**Conclusion:**

Disparities existed in this study between rural and urban areas in the practice of birth preparedness. This calls for more health education interventions to increase the practice of birth preparedness in rural areas, having an ideal birth plan, which targets health facility delivery.

## Background

Globally, women die every day from pregnancy and childbirth-related complications.^1^ Ninety-nine percent of these deaths occur in developing countries and more than half occur in sub-Saharan Africa.^1^ Every woman needs access to antenatal care in pregnancy, skilled care during childbirth, and care and support in the weeks following childbirth. The high number of maternal deaths in parts of the world shows the inequities inadequacy in access to these essential health services.^2^

Birth preparedness is a strategy that promotes the use of skilled maternal and neonatal care on the assumption that being prepared for childbirth and being ready for any complication that may arise reduces delays in receiving care and reduces maternal death.^3^ Pregnant women, their families, and communities need to plan effectively for births and deal with anticipated emergencies when they occur.

Being prepared for birth and anticipated emergencies or complications has been suggested by the World Health Organization as a comprehensive approach that reduces the three delays in care seeking during obstetric emergencies.^4^, ^5^ These delays include the delay in deciding to seek care, delay in reaching a health facility, and delay in receiving care where the client who has reached the health facility, waits to be seen by a trained health worker.^6^

Globally, an important element that links the essential tools and equipment required to render maternal health services and trained health personnel with the skills to use these tools and equipment is the place of delivery.^7^ The place of delivery has an influence on the outcome of labour and childbirth in terms of reduction in maternal and neonatal mortality; hence, a woman must make the right decision regarding her place of delivery to safeguard both maternal and child wellbeing.^8^

Generally, women have different preferences for their place of delivery,^9^ while the majority prefer to deliver in the hospital, others prefer non-facility delivery; a decision made out of existing or perceived socioeconomic status.^9^,^10^ Sub-Saharan Africa records some of the lowest facility delivery rates in the world.^9^,^11^ Health facility delivery contributes significantly to improved maternal and child health outcomes.^8^,^12^,^13^ Health facility delivery increases opportunities for the use of appropriate equipment and drugs, skilled presence at birth, and prompt referral to a higher level of care in the event of a complication or an emergency.^14^ Home births as well as other non-facility deliveries on the other hand pose high risks to the health of the mother and child.^15^-^17^ To achieve Sustainable Development Goal 3, it is required that at least 80% of all deliveries should take place in a health facility, under the supervision of a skilled health professional.^18^,^19^

In Nigeria, only about 36% of births take place in a health facility while 63% of women deliver at home.^20^ Recent estimates from the multiple indicator cluster surveys in 2016-2017, by the National Bureau of Statistics and the United Nations International Children's Emergency Fund, put the percentage of women (15-49 years old) that delivered in the health facility in Northeast Nigeria at 25.8 percent while 74 percent delivered at home.^21^ Studies have reported that women who prepared for childbirth were more likely to deliver in health facilities compared to those women who were not.^22^,^23^ Women residing close to a facility that has a skilled health worker are more likely to have facility-based delivery compared to those residing far away from a health facility without a skilled health worker.^15^,^24^,^25^

Existing studies show that the status of birth preparedness in most developing countries is less than 50%.^26^ This low status of birth preparedness and complication readiness was also evidenced in a study conducted in Northern Nigeria in 2010, 6.2% planned for skilled care at birth, 19.5% saved money, and 24.2% arranged means of transportation.^8^,^27^ A study in rural Ghana on birth location preferences of mothers and fathers revealed that preference for home birth resulted in delayed care-seeking and was associated with several cases of stillbirths and postpartum morbidities. It also revealed on the other hand that preference for facility birth resulted in early care-seeking, and possibly enabled women to avoid adverse effects of birth complications.^28^ In Sagamu, Ogun state, southwestern Nigeria, the preferred locations of delivery were government facilities (54.8%), private hospitals (24.5%), traditional birth attendants (13.5%), and spiritual healing homes (5.6%).^29^

Despite the benefits of birth preparedness in reducing the three phases of delay (in deciding to seek care, in reaching a health facility, and in receiving care)^6^ and thus reducing maternal as well as neonatal deaths and complications, few studies have assessed the association of birth preparedness with the place of delivery in rural and urban areas in Ogun State, Nigeria. Studies have also shown that disparities exist among rural and urban women in terms of the use of health facilities as places of delivery in developing countries^30^-^32^ with rural areas being the disadvantaged.^33^

The general objective of this study was to assess the practice of birth preparedness and its association with the place of delivery among women in rural and urban communities in Ogun East Senatorial District, Nigeria.

## Methodology

This study was conducted in Ogun East Senatorial District, Southwestern Nigeria. Ogun East Senatorial District has a population of 1.25 million (Census 2006) and a projected population of 1.74 million by 201634 and 1.96 million by 2020.^35^

This was a community-based comparative cross-sectional study conducted among 750 women of childbearing age, who had lived in the selected areas for at least 36 months and had at least a birth within the last 36 months regardless of birth outcome. This study excluded women who were severely ill and unable to participate in the study and those who have stayed in Ogun East Senatorial District for less than 36 months. With a power of 90% and a confidence level of 95%, the minimum sample size was obtained using the statistical formula for comparative study.^36^

The multi-stage sampling technique was used to select study participants. In stage one, the local government areas in Ogun East Senatorial District were stratified into rural and urban. Simple random sampling by balloting was used to select one rural and one urban LGA. Sagamu Local Government Area was selected as the urban local government while Odogbolu Local Government Area was selected as the rural local government. Simple random sampling by balloting was used to select one of the fifteen wards in Sagamu and Odogbolu LGAs. For Sagamu LGA, Batoro ward was selected while for Odogbolu LGA, Okun-owa ward was selected. House numbering was conducted in the selected wards in random sampling was used to select houses. The sampling interval of 5 was used in both wards after dividing the total number of houses by the sample size. The first house was selected by simple random sampling, subsequently, every 5th house was selected. In the selected houses, all households were identified and where there was more than one eligible household in the house, one of them was selected using simple random sampling by balloting. In the final stage, the eligible woman in each selected household was identified and interviewed. Where there was more than one eligible woman in the selected household, simple random sampling was used to select one of the eligible women for the interview. Houses in which there was no eligible woman were skipped.

The data collection tool was administered by an interviewer, structured questionnaire adapted from the safe motherhood questionnaire of the maternal and neonatal health program of the Johns Hopkins Program for International Education in Gynecology and Obstetrics (JHPIEGO) and the Nigeria Demographic Health Survey 2018 was used.^37^,^38^ It was pre-tested in Ake III ward, Abeokuta South LGA in Ogun Central Senatorial District. The data collection tool assessed respondents' socio-demographic information, the practice of birth preparedness during the last pregnancy, and the place of last delivery. The data were analyzed using the statistical package for Social Sciences version 22.

Place of delivery was analyzed as a binary variable with two categories: “Had facility delivery”/“did not have facility delivery”. Deliveries that took place in either a public or private health care facility were classified as “had facility delivery”. Deliveries that took place anywhere else were classified as “did not have facility delivery”. Birth preparedness was analyzed as a binary variable with two categories: “prepared” or “not prepared”. A woman was classified as “prepared” in her most recent delivery if she had done at least three of the following basic components of birth preparedness: identified a skilled birth attendant, decided on a place of delivery, saved money for delivery, identified a means of transport to a place of childbirth or identified a blood donor.^39^-^41^ A woman was classified as “not prepared” if she had done less than or equal to two of the basic components.^39^-^41^ Descriptive statistics were used to generate frequencies and proportions. T-test was used to compare the mean ages of urban and rural respondents. The chi-square test was used to test for association between independent and dependent variables at a 95% confidence interval.

## Results

Table 1 shows the socio-demographic characteristics of the respondents. In the rural area, one hundred and thirteen respondents (30.1%) were in the age group 25-29 years. Also, in the urban area, the majority of respondents (n=125, 33.3%) were in the age group 25-29 years. The difference in the mean age of rural and urban residents was not statistically significant (t = -0.840, p = 0.401). The majority of respondents in both rural and urban areas were of the Yoruba ethnic group, three hundred and twenty-nine (87.7%) and three hundred and sixty-five (97.3%) for the rural and urban residents respectively. The association between ethnicity and place of residence was statistically significant. (χ2=27.322, p=<0.001). In the rural area, fifty-seven (15.2%) respondents had no formal education as against seventeen (4.5%) in the urban area. Among the rural respondents, twenty-five (6.7%) had tertiary education as the highest educational status, while thirty-one (8.3%) respondents in the urban area had tertiary education as the highest level of education. The association between educational status and place of residence was statistically significant (χ2=45.514, p=<0.001) Among the rural residents, two hundred and forty (64%) attended ANC; this was lower compared to the three hundred and forty-seven (92.5%) of respondents in the urban area who attended ANC. The association between ANC attendance and place of residence was statistically significant (χ2=89.744, p=<0.001).

**Table 1:** Socio-demographic characteristics of respondents (N=375 per group)

Variable	Rural n (%)	Urban n (%)	Test Statistics
**Age Group (Years)**			
<24	60 (16.0)	45 (12.3)	
25-29	113 (30.1)	125 (33.3)	χ2=5.189
30-34	95 (25.3)	98 (26.1)	p=0.393
35-39	76 (20.3)	64 (17.1)	
40-44	22 (5.9)	30 (8.0)	
≥45	9 (2.4)	12 (3.2)	
**Mean**	**30.69±6.312**	**31.07±6.115**	**t= -0.840, p=0.401**
**Occupation**			
Unemployed	39 (10.4)	24 (6.4)	
Agricultural worker	9 (2.4)	3(0.8)	
Civil servant	10 (2.7)	1 (0.3)	
Trader	151 (40.3)	173 (46.1)	
Unskilled	159 (42.4)	163 (43.5)	χ2=16.367
Semi-skilled	7 (1.9)	11(2.9)	p=0.006*
**Marital status**			
Single	18 (4.8)	7 (1.9)	
Married	347 (92.5)	350 (93.3)	
Divorced	2 (0.5)	3 (0.8)	
Separated	6 (1.6)	11 (2.9)	χ2=7.190
Widowed	2 (0.5)	4 (1.1)	p=0.126
**Religion**			
Christianity	330 (88.0)	313 (83.5)	
Islam	45 (12.0)	57 (15.2)	χ2=6.861
Traditional worshipper	0 (0.0)	5 (1.3)	p=0.032*
**Ethnicity**			
Yoruba	329 (87.7)	365 (97.3)	
Igbo	9 (2.4)	2 (0.5)	
Hausa	24 (6.4)	8 (2.1)	χ2=27.322
Others	13 (3.5)	0 (0.0)	p=<0.001*
**Highest Educational status**			
No formal education	57 (15.2)	17 (4.5)	
Primary	80 (21.3)	41 (10.9)	
Secondary	213 (56.8)	286 (76.3)	χ2=45.514
Tertiary	25 (6.7)	31 (8.3)	P=<0.001*
**Parity**			
<3	190 (50.7)	204 (54.4)	χ2=1.048
≥3	185 (49.3)	171 (45.6)	p=0.306
**Attended ANC**			
Yes	240 (64)	347 (92.5)	χ2=89.744
No	135(36.0)	28 (7.5)	p=<0.001*
**Average monthly income (Naira)**			
No income	39 (10.4)	21(5.6)	
<10,000	236 (62.9)	213 (56.8)	χ2=13.553
≥10,000	100 (26.7)	141 (37.6)	p=0.001*

Mean Age= 30.88±6.213 years

* Statistically significant *t=T-test

Table 2 shows that two hundred and thirty-one (61.6%) urban respondents saved money during their last pregnancy; this was higher than the rural respondents (n=201, 53.6%) who saved money; this difference was statistically significant (p= 0.027). Generally, the most practiced basic component of birth preparedness during the last pregnancy was deciding on a place of delivery while the least practiced basic component of birth preparedness was arranging a blood donor (rural: n=115, 30.7%, urban: n=146, 38.9%). This was a statistically significant finding (p= 0.017).

**Table 2:** Practice of birth preparedness during the last pregnancy (N= 375 per group)

Variable	Rural n (%)	Urban (%)	χ2	p-value
**Saving money**	201 (53.6)	231 (61.6)	4.914	0.027*
**Identified a place of delivery**	362 (96.5)	369 (98.4)	2.646	0.104
**Arranged for a trained birth attendant**	270 (72.0)	302 (80.5)	7.541	0.006*
**Identified a specific means of emergency transport**	113 (30.1)	153 (40.8)	9.321	0.002*
**Arranged a blood donor**	115 (30.7)	146 (38.9)	5.647	0.017*
**The practice of Birth Preparedness score**				
Prepared (≥3 basic components)	245 (65.3)	274 (73.1)		
Not prepared (≤2 basic components)	130 (34.7)	101(26.9)	5.261	0.022*
**Mean**	2.83±1.326	3.19±1.317	t=-3.730	0.001*

Mean 3.01±1.333

* t=T-test

A higher proportion of urban respondents (n=274, 73.1%) were prepared, compared to rural respondents (n=245, 65.3%). A lower proportion of urban respondents (n=101, 26.9%) were unprepared compared to the proportion of rural respondents (n=130, 34.7%). The difference in the practice of birth preparedness among rural and urban respondents was statistically significant (p=0.022).

Table 3 shows that a slightly higher proportion of urban respondents (n=296, 78.9%) had health facility delivery as against two hundred and eighty-eight (76.8%) rural respondents; however, this was not statistically significant (p= 0.482). Most deliveries took place in primary health facilities in the rural area (n=104, 27.7%) while in the urban area, most deliveries took place in primary and secondary health facilities (n=110, 29.3%). The difference in the specific places of delivery among rural and urban respondents was statistically significant (p=0.030).

**Table 3:** Place of last delivery among respondents (N=375per group)

Variable	Rural n (%)	Urban n (%)	Test Statistics
**Place of last delivery**			
Health facility	288 (76.8)	296 (78.9)	
Non-health facility	87 (23.2)	79 (21.1)	χ2=0.495,p= 0.482
**The specific place of last delivery**			
Tertiary health facility	87 (23.2)	76 (20.3)	
Secondary health facility	97 (25.9)	110 (29.3)	
Primary health facility	104 (27.7)	110 (29.3)	
TBA	57 (15.2)	67 (17.9)	
At home	30 (8.0)	11 (2.9)	
Church	0 (0.0)	1 (0.3)	χ2=12.338, p=0.030*

Table 4 shows that the association between birth preparedness practice and place of delivery was statistically significant (p=<0.001) among rural and urban respondents.

**Table 4:** Association between birth preparedness practice and place of delivery. (N=375)

Variable	Rural n (%)		Urban n (%)	
	Health facility	Non-health facility	Health facility	Non-health facility
**The practice of birth preparedness**				
Prepared	242 (98.8)	3(1.2%)	273 (99.6)	1 (0.4)
Not prepared	46 (35.4)	84 (64.6)	23 (22.8)	78 (77.2)
	**χ2 (p-value)** 191.55 (<0.001)*		262.19 (<0.001)*	

## Discussion

It is vital that all pregnant women have a written plan for birth and a plan for dealing with unexpected adverse events such as complications or emergencies, that may occur during pregnancy, childbirth, or the immediate postnatal period, and should discuss and review this plan with a skilled attendant at each antenatal assessment and at least one month before the expected date of birth.^42^ A birth preparedness plan includes identification by the pregnant woman of the following elements: the desired place of birth, the preferred birth attendant, the location of the closest appropriate care facility, funds for birth-related and emergency expenses, a decision-maker during the birth process, a birth companion, a support person to look after the home and children while the woman is away, transport in the case of an obstetric emergency and identification of compatible blood donors in case of emergency.^1^

Regarding birth preparedness practice, a high proportion of respondents both in the urban and rural areas practiced birth preparedness. This is in contrast to a study carried out in Port-Harcourt, Rivers State, Nigeria where a higher proportion of respondents did not practice birth preparedness.^43^ This is also in contrast to a study in Sokoto State, Nigeria where rural respondents were noted to have had a poor practice of birth preparedness.^44^ The difference in tis this study could have been due to a higher antenatal care clinic attendance where the components of birth preparedness could have been taught. In addition, cultural and religious factors may have played a role in the difference. The findings of a higher proportion of respondents who practiced birth preparedness were also in contrast to a study in Bamenda health district Cameroon, where the practice of birth preparedness was unsatisfactory.^45^

The commonest birth preparedness practice in this study was the identification of a place of delivery. This may have been because every pregnant woman desires a place where her delivery will be taken without complications. The finding of identification of a place of delivery in this study as the commonest birth preparedness practice was in consonance with a similar study carried out in Ogun central senatorial district, Nigeria.^46^ It was also similar to the findings in Bangladesh^47^ where the commonest practice was the identification of a place of delivery and a birth attendant. However, this finding in the current study was in contrast to a study in Kano State, Nigeria where saving money was the commonest birth preparedness practice among respondents.^48^ The least birth preparedness practice in this study was identifying a blood donor or a means of obtaining safe blood. This could have been attributed to the belief system of the respondents on the fact that they wouldn't need a blood donor or blood transfusion during delivery; this finding aligned with a mixed-methods study in Ogun State Nigeria, where identifying a blood donor was also the least reported practice^46^ and also in consonance with a study carried out in India^49^ where only 15.8% of respondents arranged a blood donor.

Concerning the place of delivery, two hundred and ninety-six (78.9%) urban respondents and two hundred and eighty-eight (76.8%) rural respondents delivered in a health facility; this was higher than a study conducted in northern Nigeria by Shehu CE where a lower proportion among urban respondents (65.0%) and an even much lower proportion among rural respondents (4.7%) delivered in a health facility; which he attributed to a wide disparity in the utilization of health facilities by urban and rural respondents.^50^ The higher proportion noted in this study could have been due to the much higher utilization of health facilities.

## Conclusion

This study revealed that women in rural and urban communities of Ogun East Senatorial District, Nigeria practiced birth preparedness, though this was higher among urban dwellers than rural dwellers. The majority of rural and urban women had their last deliveries in health facilities, though the proportion of which was higher among urban than rural respondents. The practice of birth preparedness was found to influence the eventual place of delivery. More health education interventions are required to increase the practice of birth preparedness especially in rural areas, and to have an ideal birth plan, which targets a health facility delivery. Avenues for these health education interventions could be during town hall meetings, women's day programs/meetings, mass media especially the radio and television, in addition to antenatal clinic visits.

## Limitations of the study

Recall bias was a limitation of this study since it involved remembering a past event. However, this was considered minimal since giving birth is a personal experience that may not be easily forgotten.

## List of Abbreviations

ANC: Antenatal care
BUHREC: Babcock University Health Research and Ethics Committee
JHPIEGO: John Hopkins Program for International Education in Gynecology and Obstetrics
LGA: Local Government Area.

## Declarations

Ethical clearance was obtained from Babcock University Health Research Ethics Committee (BUHREC/309/20b), and the Ministry of Health, Ogun State (HPRS/381/339), Nigeria.

Verbal and written informed consent was obtained from respondents and confidentiality was ensured.
